# USF1-CHCHD4 axis promotes lung adenocarcinoma progression partially via activating the MYC pathway

**DOI:** 10.1007/s12672-022-00600-3

**Published:** 2022-12-08

**Authors:** Yuhui Zhou, Yunxia Zhao, Wei Ma, Lin Zhang, Yuanzhu Jiang, Wei Dong

**Affiliations:** 1 Department of Thoracic Surgery, Shandong Provincial Hospital Affiliated to Shandong First Medical University, 324 Jingwu Road, Jinan, 250021 People’s Republic of China; 2 Department of Neurology, Shandong Provincial Hospital Affiliated to Shandong First Medical University, Jinan, 250021 People’s Republic of China

**Keywords:** LUAD, CHCHD4, USF1, MYC pathway

## Abstract

**Background:**

This study aimed to identify genes related to lung adenocarcinoma (LUAD) and investigate the effects and molecular mechanisms of coiled-coil-helix-coiled-coil-helix domain containing 4 (CHCHD4) in the progression of LUAD.

**Methods:**

The GEPIA database was used to evaluate the differential expression of CHCHD4 and the survival data of LUAD patients compared to controls. TCGA-LUAD database, JASPAR website, and GSEA were used to analyse the relationship between CHCHD4 and the upstream stimulating factor 1 (USF1) or MYC pathways. The proliferation, apoptosis, migration, and invasion of LUAD cells were evaluated using cell counting kit-8, 5-ethynyl-2′-deoxyuridine, colony formation, flow cytometry, wound healing, and transwell assays. qRT-PCR, western blotting, and immunohistochemistry were used to detect the mRNA and protein expression, respectively. Furthermore, xenograft tumours from nude mice were used to verify the effect of CHCHD4 on LUAD in vivo.

**Results:**

CHCHD4 overexpression was found in LUAD tumor tissues and cells, and high CHCHD4 was associated with a poor prognosis. Interestingly, CHCHD4 knockdown suppressed the malignant phenotype of the LUAD cells. Moreover, we found that USF1 upregulated CHCHD4 and promoted LUAD progression. CHCHD4 knockdown also inhibited the progression of LUAD. In addition, CHCHD4 knockdown suppressed xenograft tumour growth.

**Conclusion:**

USF1-CHCHD4 axis can promote LUAD progress, which may be through activating MYC pathway.

**Supplementary Information:**

The online version contains supplementary material available at 10.1007/s12672-022-00600-3.

## Highlights

1. CHCHD4 was highly expressed in LUAD.
2. Knockdown of CHCHD4 suppressed the malignant phenotype of LUAD.
3. USF1 upregulated CHCHD4 and promoted LUAD progression.
4. Knockdown of CHCHD4 inhibited LUAD progression.

**Supplementary Information:**

The online version contains supplementary material available at 10.1007/s12672-022-00600-3.

## Background

Approximately 85% of lung cancers are non-small cell lung cancer (NSCLC), and lung adenocarcinoma (LUAD) is the most prevalent subtype [1, 2]. Despite recent advances in therapies for LUAD, the efficacy of all treatments is disappointing [3]. Therefore, to discover the new targets for the early detection and treatment of LUAD, it is necessary to conduct more in-depth studies on the pathogenesis and development of LUAD.

Coiled-coil-helix-coiled-coil-helix domain containing 4 (CHCHD4), a human homologue of yeast Mia40, regulates the import of small cysteine-containing substrates [4–6]. It has been reported, that human CHCHD4 encodes two different splicing isomers, known as variant 1 and variant 2 [7]. CHCHD4 is a key component of the disulphide relay system within the mitochondrial intermembrane space [8]. Recently, an increasing number of studies have shown that CHCHD4 is highly expressed in human cancers, and its increased expression is related to increased tumour progression, poor patient survival, and increased disease recurrence [8–10]. By analysing the GEPIA database, we showed that CHCHD4 was overexpressed in LUAD tumor tissues and that the upregulated CHCHD4 was associated with poor prognosis of LUAD patients, suggesting that CHCHD4 may be a key oncogene in LUAD.

Therefore, we explored the role of CHCHD4 and its related molecular mechanisms in LUAD. Our findings demonstrate that the upstream stimulating factor 1 (USF1)-CHCHD4 axis may promote the progression of LUAD by activating the MYC pathway, indicating that CHCHD4 might serve as a target for LUAD treatment.

## Methods

### Bioinformatics analysis

The differential expression of CHCHD4 in the tumor and adjacent normal tissue and the LUAD patient survival data were analysed using the GEPIA database (http://gepia2.cancer-pku.cn/) based on the database obtained from TCGA. Human TFDB (http://bioinfo.life.hust.edu.cn/HumanTFDB/#!/tfbs_predict) and hTFtarget (http://bioinfo.life.hust.edu.cn/hTFtarget#!/) were used to identify the transcription factors upstream of CHCHD4. Genes positively associated with CHCHD4 expression in TCGA-LUAD were analysed. Common genes were identified via the three online datasets and represented as a Venn diagram. A positive correlation between CHCHD4 and USF1 expression was identified using the TCGA-LUAD database. The binding site of USF1 in the CHCHD4 promoter region was obtained from the JASPAR website. After transfection with sh-CHCHD4, the transcriptome mRNA of A549 cells was sequenced by Novogene (Beijing, China). To evaluate the potential mechanism underlying the involvement of CHCHD4 in LUAD, we performed an enrichment analysis of pathways using the GSEA software (gene set, h.all.v7.1. symbols.gmt) based on the transcriptome mRNA sequencing results. The GESA enrichment results were plotted using R software (version 3.4.1, ggplot2 package). In addition, R software was used (gene set, h.all.v7.1. symbols.gmt; package, clusterProfiler) to conduct GSEA analysis based on TCGA-LUAD data.

### Sample collection

LUAD tissues (n = 10) and adjacent normal tissues (n = 10) were collected from patients diagnosed with LUAD who underwent surgical treatment at our hospital between August 2019 and August 2021. All the tissues were rapidly frozen and stored at 80 °C. Collection of all human clinical tissue samples was approved by the Ethics Committee of our hospital, and all samples were obtained from participants who provided written consent. Moreover, the expression of CHCHD4 was analysed in lung tissues fixed in formalin.

### Cell culture and transfection

Four human LUAD cell lines, NCI-H1975, H1299, A549, and H23, and the human bronchial epithelioid cell line 16-HBE, were supplied by Procell (Wuhan, China). All cell lines were maintained in RPMI-1640 medium (Hyclone) supplemented with 10% FBS (S660JJ, BasalMedia, Shanghai, China) and 1% streptomycin/penicillin (P1400, Solarbio, Beijing, China) in a humidified atmosphere of 5% CO2 at 37 °C. Small interfering RNAs against CHCHD4 (si-CHCHD4-1, si-CHCHD4-2, and si-CHCHD4-3) and their negative control (si-NC) were purchased from RiboBio (Guangzhou, China). Short hairpin RNAs (shRNAs) for CHCHD4 (sh-CHCHD4), and shRNA negative control (sh-NC) were obtained from Genechem (Shanghai, China). The pcDNA3.1-USF1 vector, pcDNA3.1-MYC vector and their empty vector were constructed by Tsingke Biotechnology Co., Ltd. Transfection was performed using Lipofectamine 3000 (Invitrogen, USA) according to the manufacturer's instructions.

### Cell counting Kit-8 (CCK-8) assay

Cell proliferation was assessed using the CCK-8 assay (Beyotime, Beijing, China). Briefly, cells (1 × 10^4^ cells/well) were inoculated into 96-well plates, and CCK-8 solution was added to each well. Following a 2-h incubation in 5% CO_2_ at 37 °C, the absorbance at 450 nm was determined using a microplate reader (Biorad, USA). Cell proliferation was determined at 0, 24, 48, 72, and 96 h.

### Colony formation assay

Transfected H1299 and A549 cells were seeded into a 6-well plate at 500 cells per well and cultured for two weeks. Cells were then washed with PBS, fixed using 4% paraformaldehyde (Beyotime) for 10 min at 37 °C, and stained with 5% crystal violet (Beyotime). After washing the excess staining solution with PBS, the number of colonies was counted under a light microscope.

### 5-ethynyl-2’-deoxyuridine (EdU) assay

After transfection for 48 h, H1299 and A549 cells were inoculated into 96-well plates and subsequently cultured with 50 μM EDU (RiboBio) for 120 min. The cells were then fixed with 4% paraformaldehyde for 25 min at 37 °C and treated with 0.5% Triton X-100 for 15 min. The cells were incubated with 1X Apollo reaction reagents (15 μl) for 25 min at 37 °C. After that, 4′, 6-diamidino-2phenylindole (DAPI, Beyotime) was added into each well to stain the cell nuclei for 30 min in the dark. Finally, EdU-positive cells were analysed using a fluorescence microscope.

### Flow cytometric analysis

An annexin V-FITC/PI apoptosis kit (Vazyme, Nanjing, China) was used to analyse apoptosis. Following transfection for 48 h, 5 μl annexin-FITC and 5 μl PI were added to H1299 and A549 cells for 20 min in the dark at room temperature. Apoptotic cells were analysed within one hour via using a flow cytometer (Becton Dickinson, CA, USA).

### Wound healing assay

Transfected H1299 and A549 cells (1 × 10^5^ cells/well) were inoculated into a 6-well plate and subsequently cultured to full confluence. After that, a scratched wound was created using a pipette tip, and PBS was applied to remove cell debris. After culturing for 24 h, the migration process was imaged using an inverted microscope.

### Transwell assay

After transfection for 48 h, approximately 2 × 10^4^ H1299 and A549 cells were suspended in RPMI-1640 medium and inoculated into the Transwell upper chamber (Corning) with or without Matrigel. RPMI-1640 medium (500 μl) containing 10% FBS was then added to the lower chamber. After incubation at 37 °C for 24 h, cells on the bottom surface of the transwell filter were fixed with 4% paraformaldehyde (Beyotime) and stained with 0.1% crystal violet (Beyotime). Finally, migrated and invasive cells were counted under a microscope.

### Dual luciferase reporter assay

A mutant CHCHD4 promoter fragment, including a putative USF1 binding site or the wild-type fragment, was synthesised and inserted into the pGL3 vector. The promoter sequence was synthesized by Genscript Biotech Corporation. Wild-type and mutated USF1 promoters were synthesised separately. Wild-type and mutant USF1 promoter vectors were identical. pGL3, pGL3-CHCHD4, pcDNA3.1-USF1, and pcDNA3.1 were transfected into HEK-293 T cells using Lipofectamine 3000 (Invitrogen). After culturing at 37 °C for 48 h, the cells were detected using a dual-luciferase reporter assay system (#E1910, Promega, USA) according to the manufacturer’s instructions.

### RNA extraction and quantitative reverse transcription (qRT-PCR)

Total RNA was freshly extracted from H1299 and A549 cells using TRIzol reagent (Thermo Fisher). Complementary DNA (cDNA) was synthesised using a PrimeScript RT Reagent Kit (Takara, Dalian, China). qRT-PCR was conducted using SYBR Premix Ex Taq (Takara) on a CFX96 Fast Real-Time PCR System (Biorad, USA). The primer sequences, supplied by Tsingke (Beijing, China), were as follows: CHCHD4 (F),5′- A CTATTGCCGGCAGGAAGGG -3′, (R):5′- TGGCAGTATCAATCCATGCTC -3′; USF1 (F):5′-ATGAAGGGGCAGCAGAAAACA-3′, (R):5′-TGTTTTCTGCTGCCCCTTCAT-3′; MYC (F):5′-TCTGCTCGCCCTCCTACGTTG-3′, (R):5′- CCTCTGGCGCTCCAAGACGTT-3′; GAPDH (F):5′-CCATGGGGAAGGTGAAGGTC-3′, (R):5′-TCGCCCCACTTGATTTTGGA-3′.

### Western blot

Total protein was extracted from cells, lung tissues of patients, and xenograft tumour tissues of mice using RIPA lysis buffer (Elabscience). Total protein was subjected to 10% SDS-PAGE, and then transferred to polyvinylidene difluoride membranes. Polyvinylidene difluoride membranes were blocked with 5% non-fat milk in TBST for 1 h at 37 °C. After blocking, the membrane was incubated with primary antibodies (CHCHD4, cat no. 21090-1-AP, Proteintech, Wuhan, China; Bcl-2, cat no. 26593-1-AP, Proteintech; Cleaved-PARP, cat no. #5625, Cell Signalling, USA; Bax, cat no. 50599-2-Ig, Proteintech; E-cadherin, cat no. A20798, Abclonal, Wuhan, China; N-cadherin, cat. ab280375, Abcam, USA; vimentin, cat no. 10366-1-AP, Proteintech; MYC, cat no. 10828-1-AP, Proteintech, USA; Snai1, cat no. 13099-1-AP, Proteintech; Snai2, cat no. 12129-1-AP, Proteintech; and Twist1, cat no. A7314, Abclonal,; GAPDH, cat no. 10494-1-AP, Proteintech) overnight at 4 °C. Membranes were then incubated with horseradish peroxidase-conjugated goat polyclonal anti-rabbit IgG secondary antibodies (cat. no. ab150077; Abcam) for 1 h at room temperature. The target bands were visualised using a chemiluminescence kit (Beyotime).

### Xenograft tumors in nude mice

Five-week-old male BALB/c nude mice were obtained from GemPharmatech Co. Ltd. (Jiangsu, China). A549 cell suspensions transfected with sh-NC or sh-CHCHD4 (5.0 × 10^7^ cells/ml, 100 μl) were subcutaneously injected into the left dorsal flanks of each mouse. Mice was randomly divided into two groups (n = 5/group): sh-NC and sh-CHCHD4. Tumour volumes were recorded every three days from the 10^th^ to 30^th^ day. Thirty days after injection, xenograft tumours were collected and fixed in paraformaldehyde. The maximal tumor size/burden approved by your IRB was not exceeded. The experimental protocol of our study was performed in accordance with the Guide for the Care and Use of Laboratory Animals and approved by Shandong Provincial Hospital Affiliated to Shandong First Medical University.

### Terminal dexynucleotidyl transferase-mediated dUTP nick end labelling (TUNEL) assay

The TUNEL assay kit (Elabscience) was used to evaluate cell apoptosis in vivo, in accordance with the manufacturer’s instructions. Apoptotic cells in lung tissues were observed under a fluorescence microscope.

### H&E staining and Immunohistochemistry

Xenograft tumours and human lung tissues were embedded in paraffin and sectioned into 5-μm thickness after embedding. For H&E staining, the sections were double stained with haematoxylin and eosin, and histopathological changes were analysed under a light microscope.

For immunohistochemistry, the sections were blocked with 5% goat serum (ZLI-9021, ZSGB-BIO, Beijing, China) for 60 min and then incubated with primary antibodies against CHCHD4 (CHCHD4, cat no. 21090-1-AP, Proteintech), ki67 (cat no. 12075, Cell Signalling) at 4 °C overnight. After incubation with an HRP-conjugated secondary antibody and staining with 3′,3-diaminobenzidine (DAB; ZLI-9017, ZSGB-BIO), the slides were observed and photographed under a light microscope.

### Statistical analysis

SPSS22.0 (SPSS, Chicago, IL, USA) was used for statistical analyses. Data are expressed as means ± standard deviation (SD). The Mann–Whitney U-test was used to compare differences between two groups. In all analyses, the significance level was set at P < 0.05. Each experiment was repeated thrice.

## Results

### CHCHD4 overexpression was found in LUAD tissues and cells and was associated with a poor prognosis in LUAD patients

**Fig. 1:**
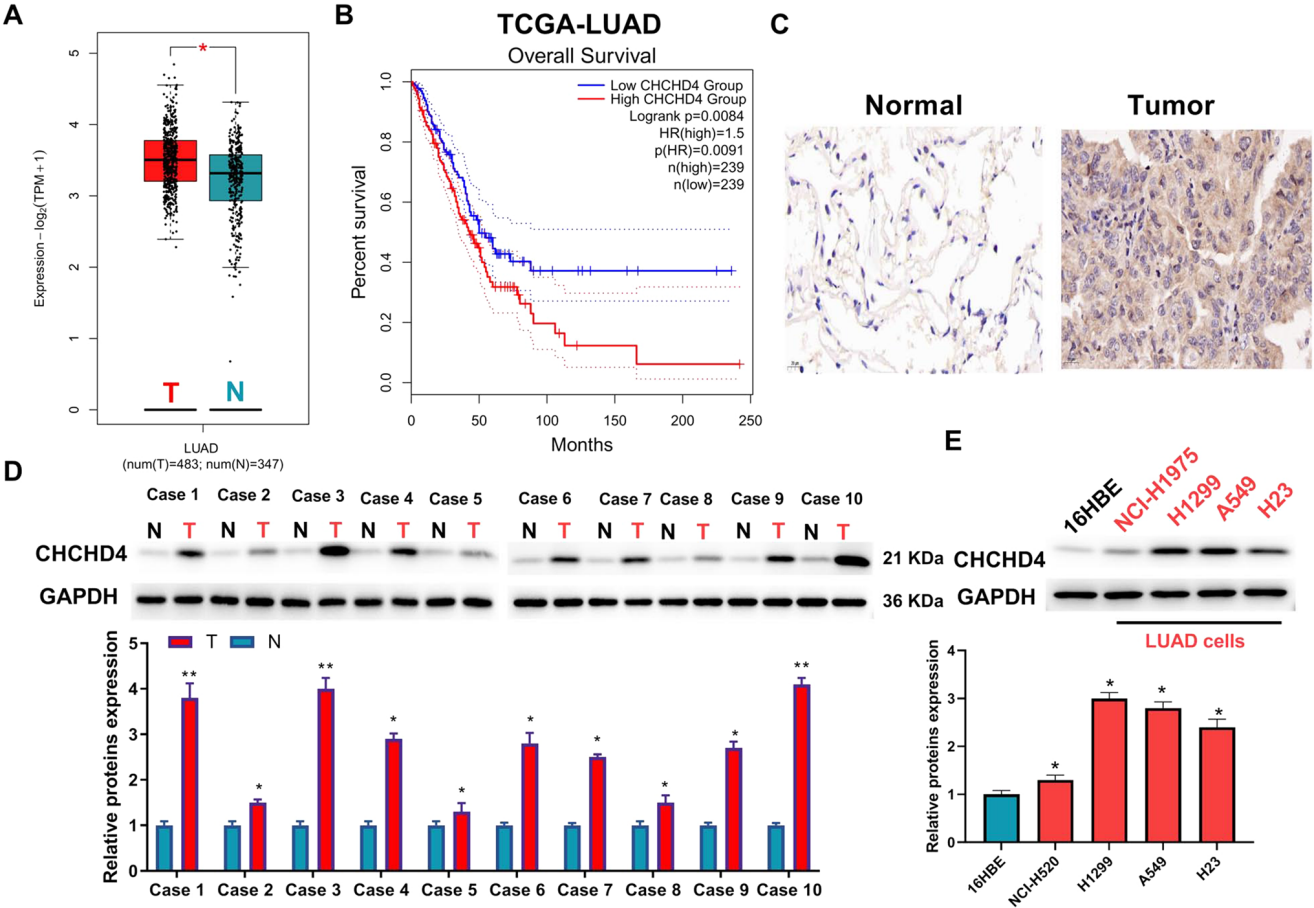
CHCHD4 overexpression was found in LUAD patients and cells and was closely associated with a poor prognosis. **A** CHCHD4 differential expression in GEPIA database. **B** The survival curves of LUAD patients from GEPIA database. **C** Immunohistochemistry Assessment of CHCHD4 expression in LUAD tissues using immunocytochemistry. Assessment of the protein levels of CHCHD4 in the tissues of patients with LUAD (**D**) and LUAD cells (**E**) using western blot. ^*^P < 0.05, ^**^P < 0.01

By analysing the GEPIA database, we found that CHCHD4 is overexpressed in patients with LUAD (Fig. 1A). Furthermore, data from the GEPIA database revealed that the upregulation of CHCHD4 was related to the poor prognosis of LUAD patients (Fig. 1B). Immunohistochemistry and western blotting demonstrated that its high expression in LUAD tissues (Fig. 1C–D and Figure S1). Furthermore, CHCHD4 was overexpressed in LUAD cells (Fig. 1E).

### Knockdown of CHCHD4 suppressed proliferation and accelerated apoptosis in A549 and H1299 cells

**Fig. 2:**
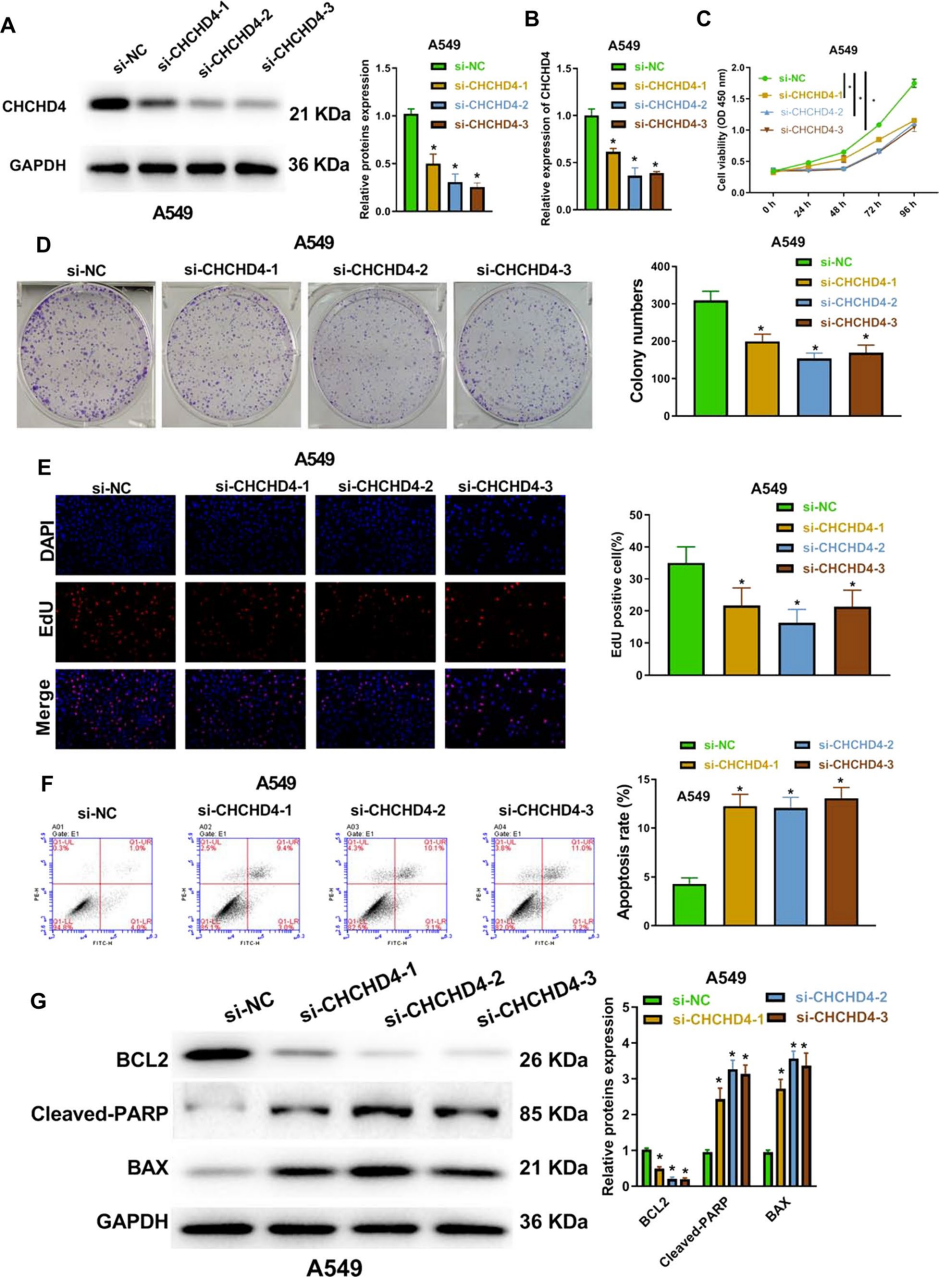
Knockdown of CHCHD4 repressed cell proliferation and accelerated apoptosis in LUAD cells. After si-CHCHD4-1, si-CHCHD4-2 and si-CHCHD4-3 were transfected into A549 cell, CHCHD4 expression levels were evaluated using western blot (**A**) and qRT-PCR (**B**); cell viability was examined using CCK-8 assay (**C**); cell colonies were analysed with colony formation assay (**D**); proliferation was tested using EdU assay (**E**); apoptosis was assessed using FITC-Annexin V/PI apoptosis detection kit (**F**); the levels of apoptosis-related proteins were evaluated using western blot (**G**). ^*^P < 0.05

After transfection with si-CHCHD4-1, si-CHCHD4-2, and si-CHCHD4-3, the protein and mRNA levels of CHCHD4 were significantly decreased (Fig. 2A–B and Figure S2A-B). Moreover, the proliferation of A549 and H1299 cells was notably inhibited after transfection with si-CHCHD4-1, si-CHCHD4-2, and si-CHCHD4-3, which was shown using the CCK-8, colony formation, and EdU assays (Fig. 2C–E and Figure S2C-E). In contrast, CHCHD4 knockdown significantly increased the number of dead A549 and H1299 cells (Fig. 2F and Figure S2F). As seen in Fig. 2G and S2G, knockdown of CHCHD4 elevated Bax and cleaved PARP levels, but reduced Bcl-2 levels, which further showed that knockdown of CHCHD4 promoted the apoptosis of LUAD cells.

### Knockdown of CHCHD4 suppressed the migration and invasion of A549 and H1299 cells

**Fig. 3:**
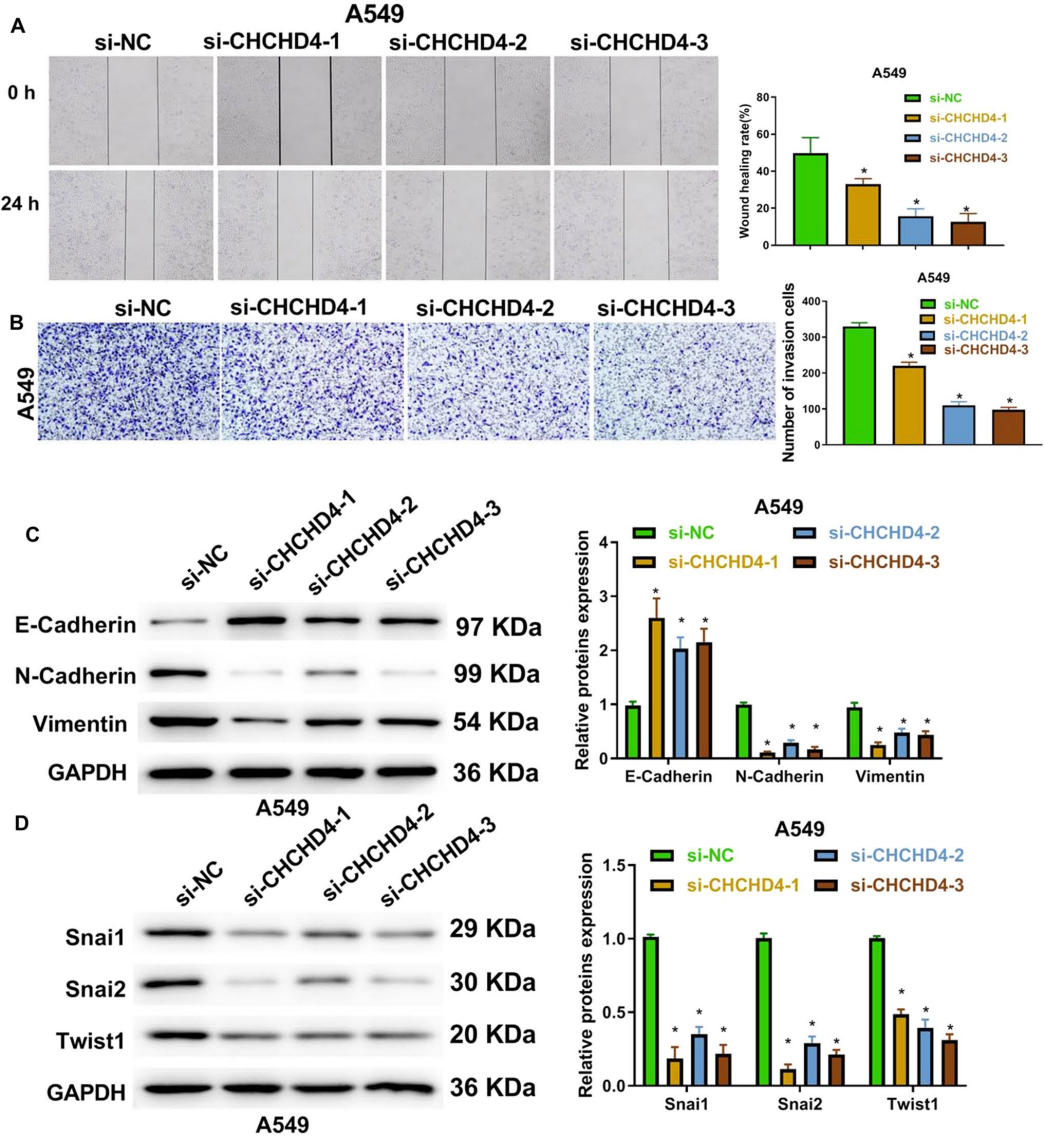
Knockdown of CHCHD4 suppressed migration and invasion of LUAD cells. After si-CHCHD4-1, si-CHCHD4-2 and si-CHCHD4-3 were transfected into A549 cells, migration was assessed using a wound healing assay (**A**); cell invasion (**B**) was examined using transwell assay; EMT-related proteins were analysed using western blot (**C**–**D**). ^*^P < 0.05

Through wound healing and transwell assays, we showed that CHCHD4 silencing notably repressed the migration and invasiveness of LUAD cells (Fig. 3A-B and Figure S3A-B). Epithelial-mesenchymal-transition (EMT) plays a crucial role in the migration and invasion of cancer cells [11]. Thus, we examined EMT-related protein levels using western blotting and found that knockdown of CHCHD4 reduced the expression of N-cadherin, vimentin, Snai1, Snai2, and Twist1, but increased E-cadherin expression in A549 and H1299 cells (Fig. 3C–D and Figure S3C-D). These results revealed that CHCHD4 silencing repressed the migration and invasion of LUAD cells.

### USF1 upregulated CHCHD4 and promoted the progression of LUAD

**Fig. 4:**
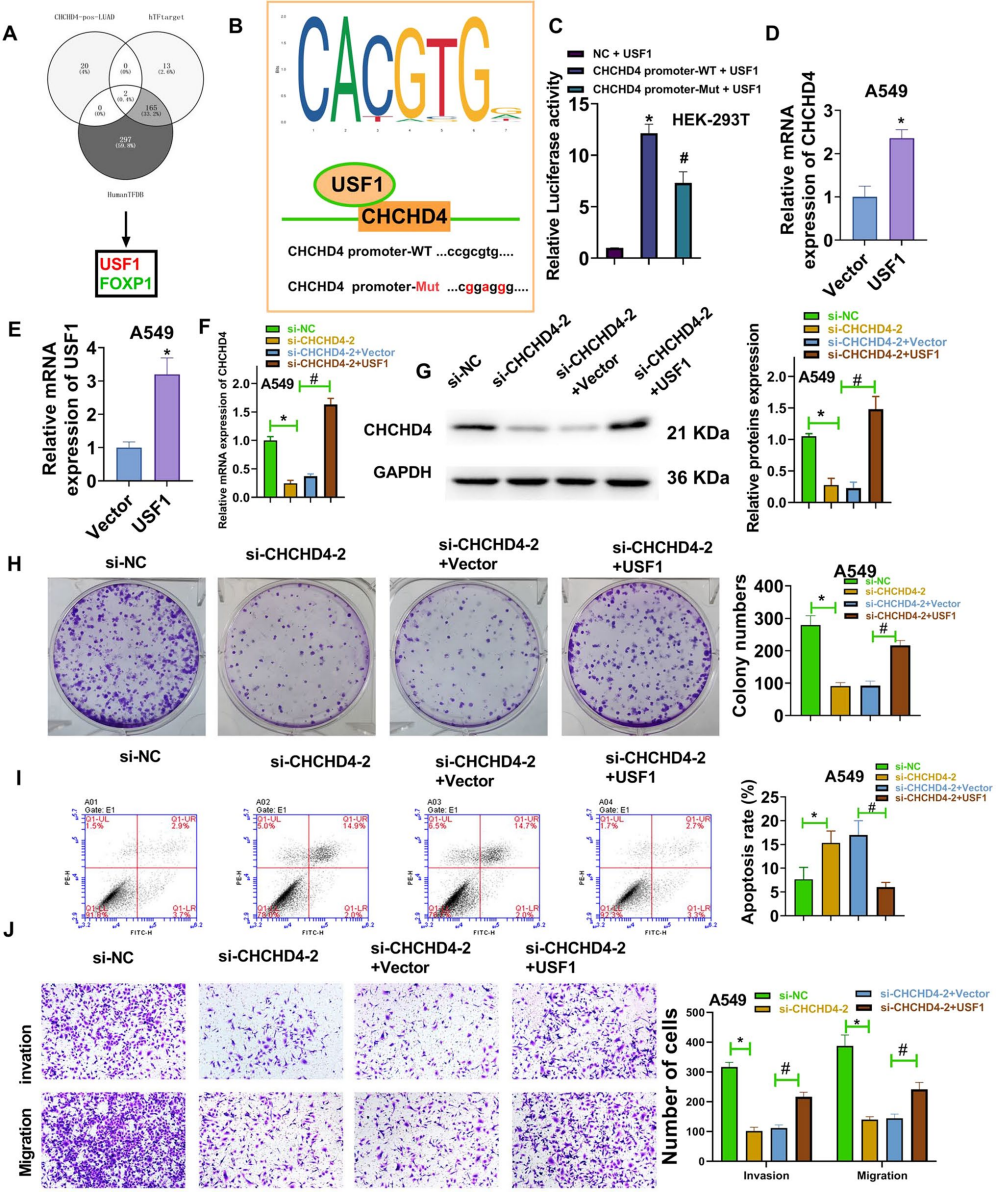
USF1 upregulated CHCHD4 and promoted the progression of LUAD. **A** Venn chart identified 2 genes (*USF1* and *FOXP1*) via analysing the human TFDB, hTFtarget and the genes positively associated with CHCHD4 expression in TCGA-LUAD. **B** The JASPAR website was used to identify the binding sites of USF1 in the CHCHD4 promoter region. **C** Luciferase activities were evaluated via performing dual luciferase reporter assay. After pcDNA3.1-USF1 vector was transfected into A549 cells, the mRNA levels of CHCHD4 (**D**) and USF1 (**E**) were determined using qRT-PCR. After pcDNA3.1-USF1 vector and si-CHCHD4-2 were co-transfected into A549 cells, the expression of CHCHD4 was measured using qRT-PCR and western blot (**F**–**G**); cell colonies were analysed using a colony formation assay (**H**); apoptosis was assessed using FITC-Annexin V/PI apoptosis detection kit (**I**); migration and invasion were tested using a transwell assay (**J**). ^*^P < 0.05, ^#^P < 0.05

The Venn chart represents two genes (*USF1* and *FOXP1*) from the human TFDB, hTFtarget, and genes positively associated with CHCHD4 expression in TCGA-LUAD (Fig. 4A). The binding site of USF1 on the CHCHD4 promoter region was obtained using the JASPAR website (Fig. 4B). The specific coordinates of the promoter region site are chr3:14,113,861–14,113,867 (reverse strand). To further verify that USF1 mediates the upregulation of CHCHD4, a dual luciferase reporter assay was performed. As seen in Fig. 4C, luciferase activity was increased in the CHCHD4 promoter-WT + USF1 group compared to the NC + USF1 group. Meanwhile, luciferase activity was decreased in the CHCHD4 promoter-MUT + USF1 group compared with that of the CHCHD4 promoter-WT + USF1 group. Moreover, after A549 cells were transfected with the pcDNA3.1-USF1 vector, CHCHD4 expression was significantly elevated (Fig. 4D), further indicating that CHCHD4 is positively correlated with USF1 expression in LUAD cells. Subsequently, the transfection efficiency of the pcDNA3.1-USF1 vector in A549 cells was analysed using qRT-PCR (Fig. 4E). To evaluate the levels of CHCHD4 in cells co-transfected with CHCHD4 siRNA and pcDNA3.1-USF1 vector, we performed RT qPCR and western blot analyses. The results showed that the expression of CHCHD4 decreased significantly after cells were transfected with si-CHCHD4-2. After co-transfection with si-CHCHD4-2 and pcDNA3.1-USF1, the expression levels of CHCHD4 increased (Fig. 4F–G). Furthermore, Fig. 4H–J shows that upregulated USF1 significantly reversed the inhibitory effect of CHCHD4 knockdown on the malignant phenotypes of A549 cells. All these results show that USF1 can upregulate CHCHD4 and promote the progression of LUAD.

### Knockdown of CHCHD4 inhibited the progression of LUAD

After transfection with sh-CHCHD4, the transcriptome mRNA sequencing of A549 cells was sequenced by Novogene (Beijing, China) (Figure S4A-S4B). Moreover, the GSEA analysis of sequencing data showed that MYC signal pathway was significantly inhibited after CHCHD4 knockdown. (Figure S4C and S4E). In addition, the GSEA analysis of TCGA-LUAD data confirmed that CHCHD4 expression was positively related to MYC signalling (Figure S4D), which was consistent with the enriched pathway of sequencing results. Furthermore, the results of the sequencing data also showed that MYC expression in sh-CHCHD4 group decreased significantly (Figure S4F).

**Fig. 5:**
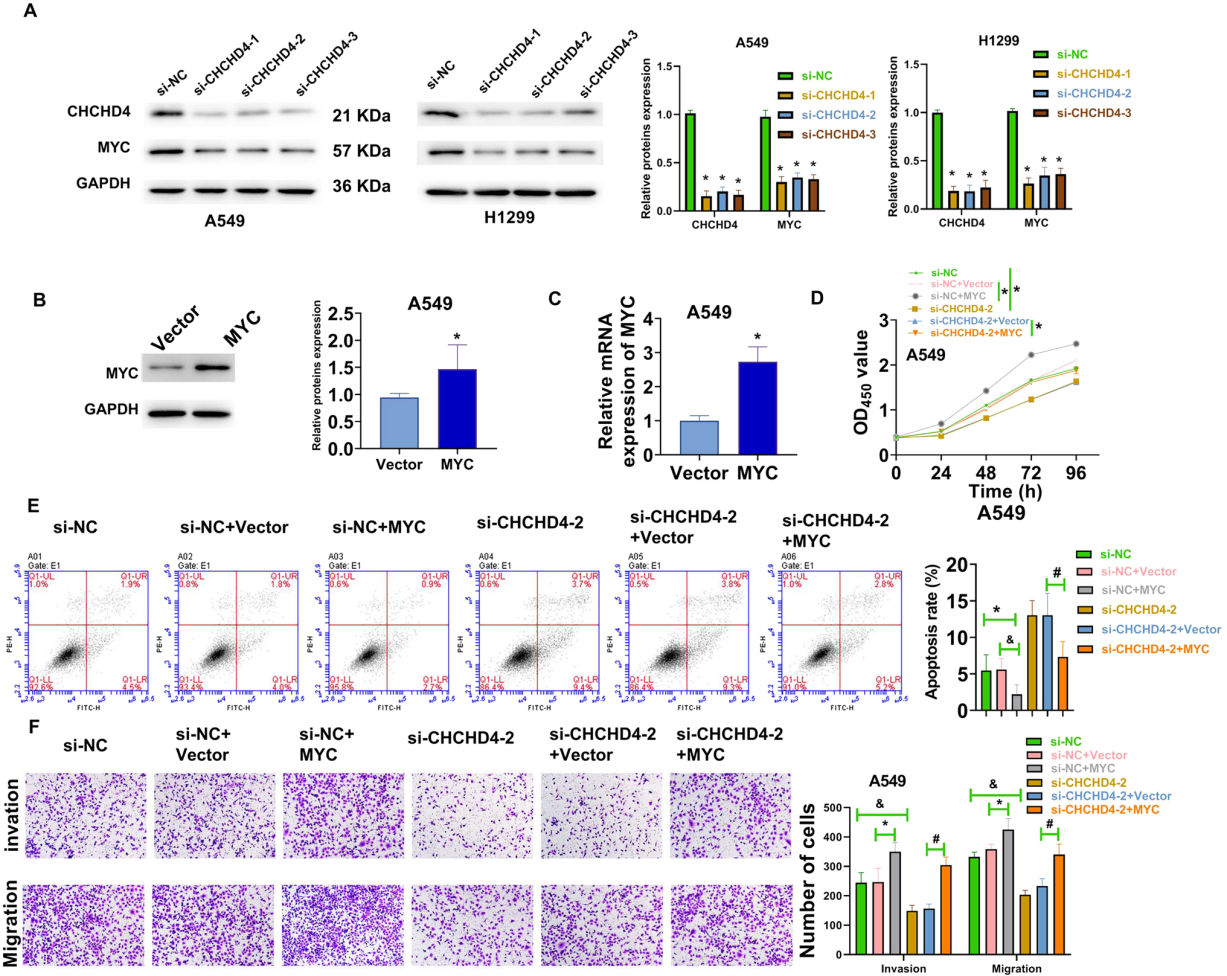
Knockdown of CHCHD4 inhibited the progression of LUAD via suppressing the MYC pathway. **A** After si-CHCHD4-1, si-CHCHD4-2, and si-CHCHD4-3 were transfected into A549 and H1299 cells, the protein levels of CHCHD4, and MYC were determined using western blot. **B**–**C** After pcDNA3.1-MYC vector was transfected into A549 cells, MYC expression was determined using western blot and qRT-PCR. After pcDNA3.1-MYC vector and si-CHCHD4-2 were co-transfected into A549 cells, cell viability was analysed using the CCK-8 assay (**D**); apoptosis was assessed using FITC-Annexin V/PI apoptosis detection kit (**E**); migration and invasion were examined by applying the transwell assay (**F**). ^*^P < 0.05, ^#^P < 0.05, ^&^P < 0.05

In vitro experiments, CHCHD4 knockdown significantly reduced the expression of CHCHD4 and MYC (Fig. 5A). The transfection efficiency of pcDNA3.1-MYC vector in A549 cells was analysed using western blotting and qRT-PCR (Fig. 5B–C). Subsequently, to further verify the relationship between CHCHD4 and the MYC pathway, rescue experiments were performed using the pcDNA3.1-MYC vector. The results of CCK-8, flow cytometry, and transwell assays demonstrated that MYC overexpression notably reversed the inhibitory effect of CHCHD4 knockdown on the malignant phenotype of A549 cells (Fig. 5D–F). Together, these results indicated that knockdown of CHCHD4 may inhibit the progression of LUAD partially via suppressing the MYC pathway.

### Knockdown of CHCHD4 suppressed the growth of xenograft tumors in nude mice

**Fig. 6:**
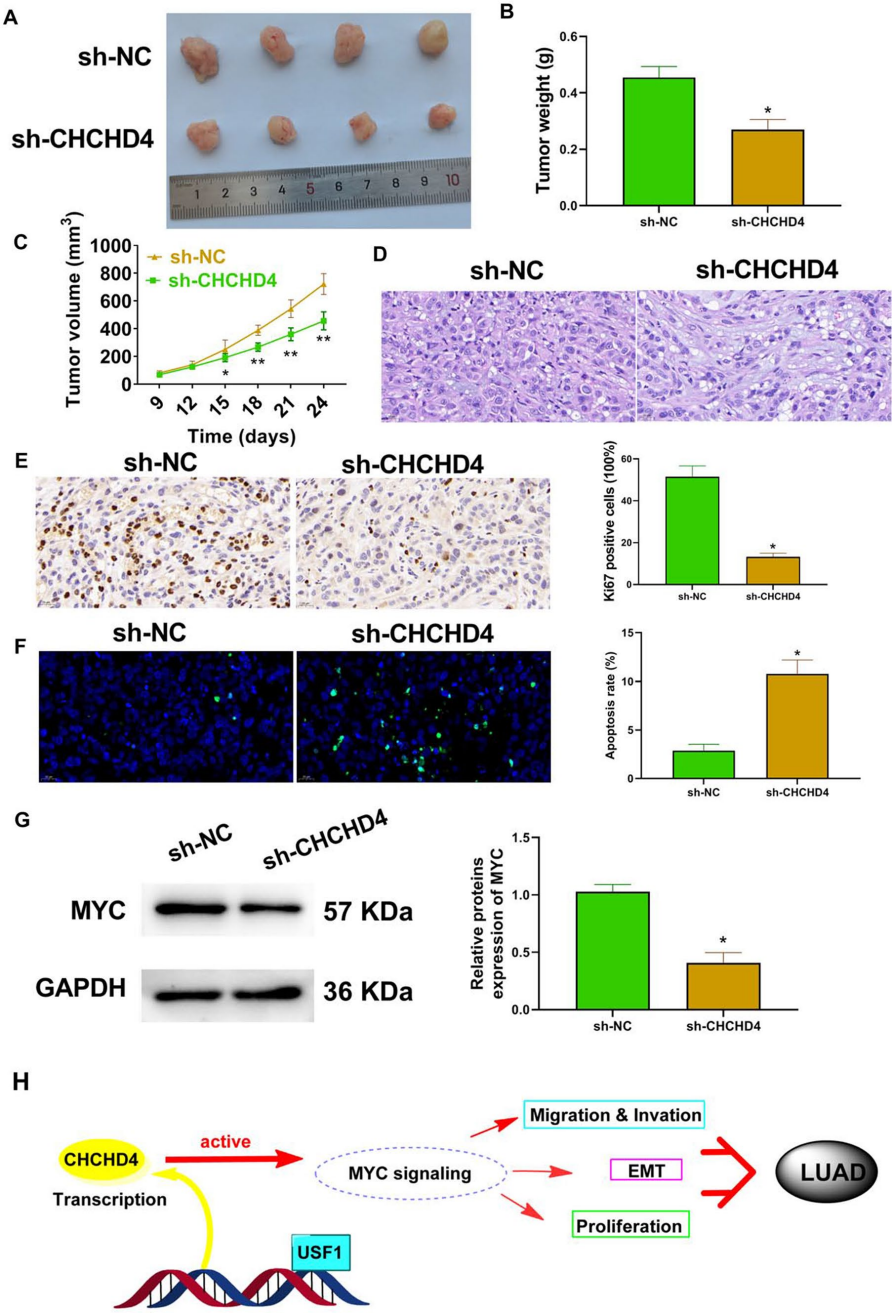
knockdown of CHCHD4 suppressed the growth of xenograft tumours in nude mice by inhibiting the MYC pathway. After A549 cells treated with sh-CHCHD4 were injected into each mouse, **A** the xenograft tumour, the tumour weight (**B**) and volume (**C**) were pictured and examined; the histopathological changes in tumour tissues were observed using HE staining (**D**); the protein levels of Ki67 in tumour tissues were evaluated using immunohistochemistry (**E**); apoptosis in tumour tissues was assessed using the TUNEL assay (**F**); MYC expression in tumour tissues was examined using western blot (**G**). **H** Proposed model of USF1-CHCHD4 axis modulating LUAD. ^*^P < 0.05

The results of the in vivo study verified that knockdown of CHCHD4 reduced tumour weight and volume (Fig. 6A–C). In addition, decreased tumour cell density was observed in the sh-CHCHD4 group (Fig. 6D). Immunohistochemistry data confirmed that the levels of KI-67 in the sh-CHCHD4 group were lower than that in the sh-NC group (Fig. 6E). Meanwhile, apoptosis was notably elevated after CHCHD4 knockdown (Fig. 6F). Furthermore, CHCHD4 knockdown significantly reduced MYC expression in xenograft tumours (Fig. 6G).

## Discussion

Recently, based on the concept of "precision medicine", advances in targeted therapy have notably improved the survival of patients with LUAD [12]. By analysing the GEPIA database, CHCHD4 was found to be overexpressed in patients with LUAD and the upregulated CHCHD4 was associated with a poor prognosis. Our study also confirmed that CHCHD4 was highly expressed in LUAD tissues and cells. Furthermore, knockdown of CHCHD4 inhibited the malignant phenotypes of LUAD cells and xenograft tumour growth, suggesting that CHCHD4 might be a key oncogene in LUAD.

Thomas et al. found that CHCHD4 is crucial for regulating intracellular oxygenation, mitochondrial localization and morphology [13]. Furthermore, pVHL can promote the expression of CHCHD4 in renal cell carcinoma cells. By using the JASPAR website, the binding sites of USF1 in the CHCHD4 promoter region were identified. Moreover, USF1 was verified to mediate the upregulation of CHCHD4 [14]. USF1, a common transcription factor in mammalian cells, can bind to the E-box motif in the promoter regions of many genes [15–17]. USF1 is reported to regulate the expression of genes associated with lipid and carbohydrate homeostasis [16, 17]. Increasing evidence has demonstrated that USF1 plays an important role in many cancers, including gastric cancer, glioma, and melanoma [18–20]. Interestingly, as a transcriptional regulator of UGT1A3, USF1 can promote LUAD progression by modulating the neurotrophin pathway [21]. Moreover, Chen et al. have reported that miR-210-3p could facilitate lung cancer progression by impairing the USF1-mediated expression of PCGF3 [22]. In this study, we found that USF1 overexpression abolished the inhibitory effect of CHCHD4 knockdown on the malignant phenotype of A549 cells, indicating that USF1 can promote the progression of LUAD by upregulating CHCHD4.

Yang et al. found that the increased expression of CHCHD4 in human tumors is related to the characteristics of hypoxia gene, and the overexpression of CHCHD4 protein in tumor cells enhances HIF-1α protein stability in hypoxic conditions [8]. In our study, GSEA analysis of transcriptome sequencing data showed that CHCHD4 expression was positively related to MYC signalling in LUAD. MYC is one of the most widely studied oncogenes involved in the growth, progression, and maintenance of various cancer types [23, 24]. In normal cells, MYC activity is rigorously modulated at both transcriptional and post-transcriptional levels [25]. Abnormal MYC expression is the most common abnormality in malignant tumours [26, 27]. In addition, MYC oncoprotein expression is thought to be related to the output of major cellular processes including proliferation, apoptosis, differentiation, and metabolism [25]. An increasing number of studies have reported that some genes promote cancer progression by activating the MYC pathway [28–30]. For example, LCAT3 plays an oncogenic role in lung cancer by binding to FUBP1 to activate MYC [31]. Wei et al. [32] showed that LPCAT1 promotes brain metastasis of LUAD by upregulating PI3K/AKT/MYC signalling. Our study demonstrated that MYC overexpression abolished the inhibitory effect of CHCHD4 knockdown on the malignant phenotype of A549 cells. Additionally, CHCHD4 knockdown suppressed the growth of xenograft tumours.

In the present study, USF1-CHCHD4 axis may promote the progression of LUAD by activating the MYC pathway (Fig. 6H). On the one hand, overexpression of USF1 in A549 can promote the expression of CHCHD4. These results provide a reference for the development of CHCHD4 inhibitors in the future. On the other hand, our findings indicate that knockdown of CHCHD4 may inhibit the progression of LUAD by suppressing the MYC pathway. Therefore, CHCHD4 may serve as a potential target for the treatment of LUAD. However, LUAD is a very complex heterogeneous disease. In this study, we only got the above conclusions in Xenograft tuners and cell experiments, which is the main limitation of this study. Next, we will expand the clinical sample size and establish Patient derived xenograms (PDXs) model to further verify the results of this study.

## Supplementary Information

**Supplementary material 1: Figure S1.** Immunohistochemistry detected CHCHD4 expression in LUAD tissues

**Supplementary material 2: Figure S2.** Knockdown of CHCHD4 repressed cell proliferation and accelerated cell apoptosis in LUAD. After si-CHCHD4-1, si-CHCHD4-2 and si-CHCHD4-3 were transfected into H1299 cell, CHCHD4 expressions were evaluated applying western blot (A) and qRT-PCR (B); cell viability was detected using CCK-8 assay (C); the cell colony was analyzed with colony formation assay (D); the proliferation was tested using EdU assay (E); the apoptosis was assessed using FITC-Annexin V/ PI apoptosis detection kit (F); apoptosis-related proteins were evaluated via performing western blot (G). ^*^P < 0.05

**Supplementary material 3: Figure S3.** Knockdown of CHCHD4 suppressed cell migration and invasion in LUAD. After si-CHCHD4-1, si-CHCHD4-2 and si-CHCHD4-3 were transfected into H1299 cell, the migration was assessed using wound healing assay (A); cell invasion (B) was tested using transwell assay; EMT-related proteins were measured using western blot (C-D). ^*^P < 0.05

Supplementary material 4: **Figure S4.** the transcriptome mRNA sequencing results of A549 cells transfected with sh-NC and sh-CHCHD4 respectively. (A and B) The transcriptome mRNA sequencing of A549 cells transfected with sh-CHCHD4 was sequenced by Novogene. (C) The data of GSEA analyses confirmed that CHCHD4 positively corelated with MYC signalling in A549 cells based on the results of transcriptome mRNA sequencing. (D) The data of GSEA confirmed that CHCHD4 positively corelated with MYC signalling in LUAD based on the TCGA-LUAD data. (E) MYC pathway was significantly inhibited after knockdown of CHCHD4 according to sequencing results. (F) The transcriptome mRNA sequencing results showed that MYC expression in sh-CHCHD4 group was significantly reduced. ^*^P < 0.05

## Data Availability

The datasets used and/or analyzed during the current study available from the corresponding author on reasonable request.

## Abbreviations

CHCHD4: Coiled-coil-helix-coiled-coil-helix domain containing 4
LUAD: Lung adenocarcinoma
NSCLC: Non-small cell lung cancer
