# Supplementary figures and images for: USF1-CHCHD4 axis promotes lung adenocarcinoma progression partially via activating the MYC pathway
Source: Discov Oncol. 2022 Dec 8;13:136. doi: 10.1007/s12672-022-00600-3 (PMC9732179; doi:10.1007/s12672-022-00600-3)

**Case2**

**Normal**

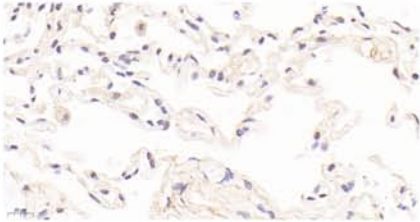

**Tumor**

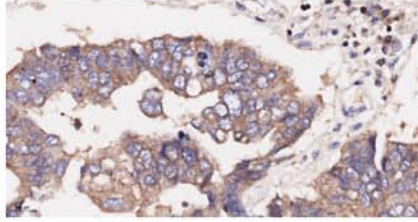

**Case3**

**Normal**

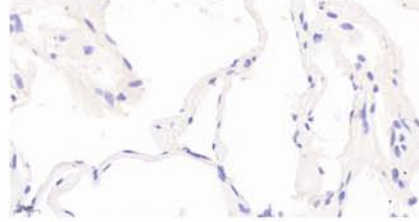

**Tumor**

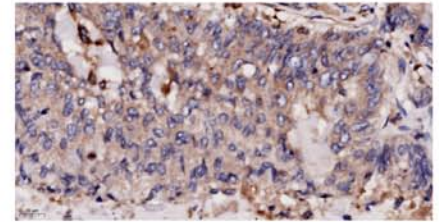

**Case4**

**Normal**

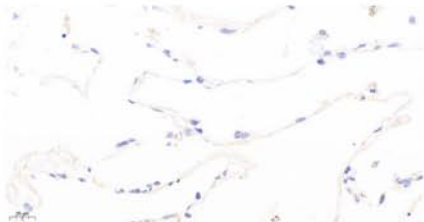

**Tumor**

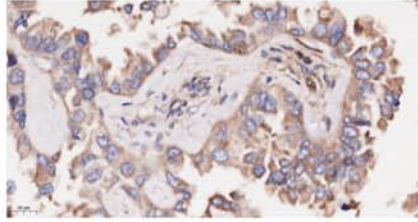

**Case5**

**Normal**

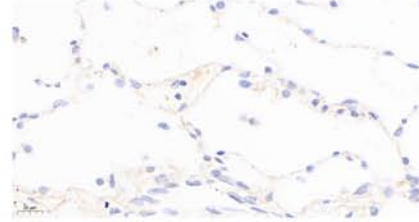

**Tumor**

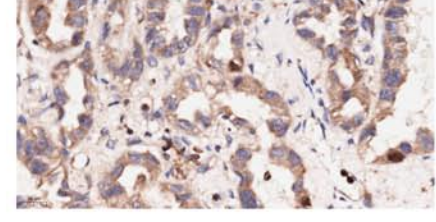

**Case6**

**Normal**

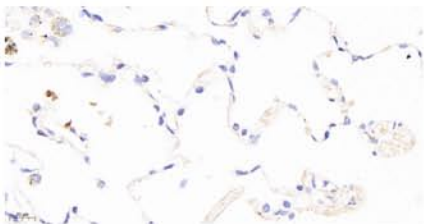

**Tumor**

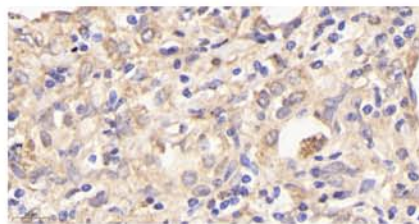

Supplement: Supplementary file 1 — Supplementary material 1: Figure S1. Immunohistochemistry detected CHCHD4 expression in LUAD tissues [file 12672_2022_600_MOESM1_ESM.pdf]

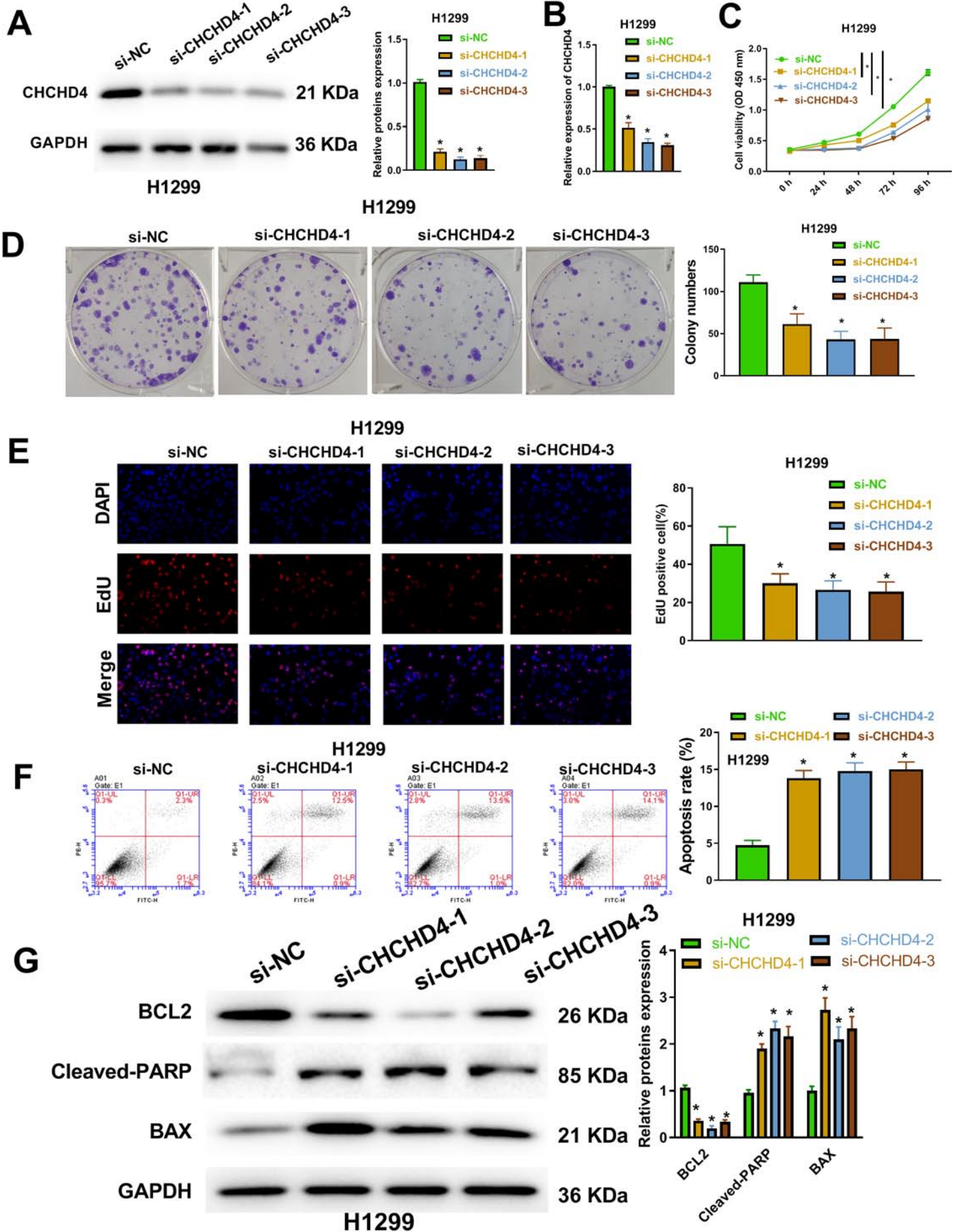

Supplement: Supplementary file 2 — Supplementary material 2: Figure S2. Knockdown of CHCHD4 repressed cell proliferation and accelerated cell apoptosis in LUAD. After si-CHCHD4-1, si-CHCHD4-2 and si-CHCHD4-3 were transfected into H1299 cell, CHCHD4 expressions were evaluated applying western blot (A) and qRT-PCR (B); cell viability was detected using CCK-8 assay (C); the cell colony was analyzed with colony formation assay (D); the proliferation was tested using EdU assay (E); the apoptosis was assessed using FITC-Annexin V/ PI apoptosis detection kit (F); apoptosis-related proteins were evaluated via performing western blot (G). *P < 0.05 [file 12672_2022_600_MOESM2_ESM.pdf]

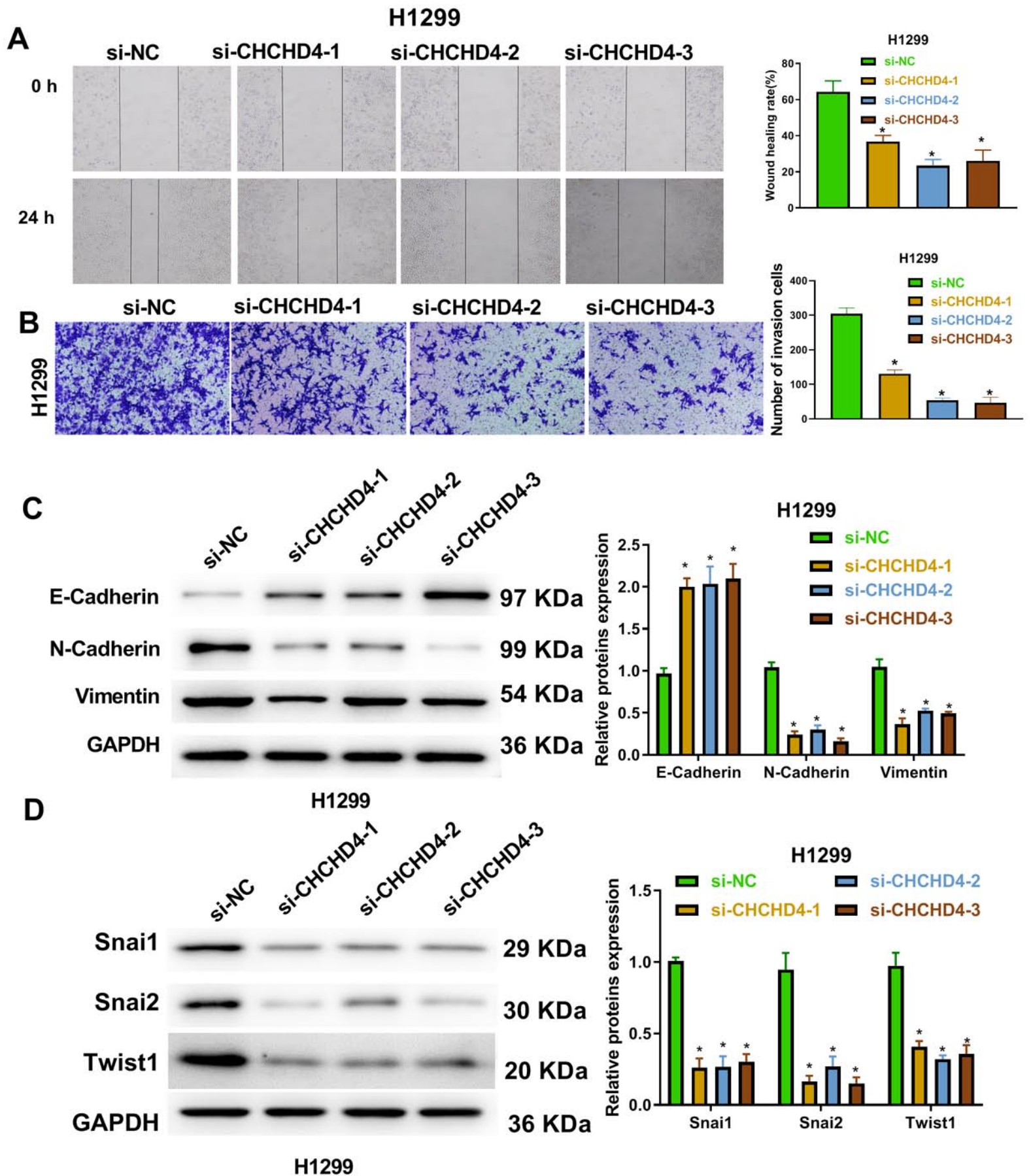

Supplement: Supplementary file 3 — Supplementary material 3: Figure S3. Knockdown of CHCHD4 suppressed cell migration and invasion in LUAD. After si-CHCHD4-1, si-CHCHD4-2 and si-CHCHD4-3 were transfected into H1299 cell, the migration was assessed using wound healing assay (A); cell invasion (B) was tested using transwell assay; EMT-related proteins were measured using western blot (C-D). *P < 0.05 [file 12672_2022_600_MOESM3_ESM.pdf]

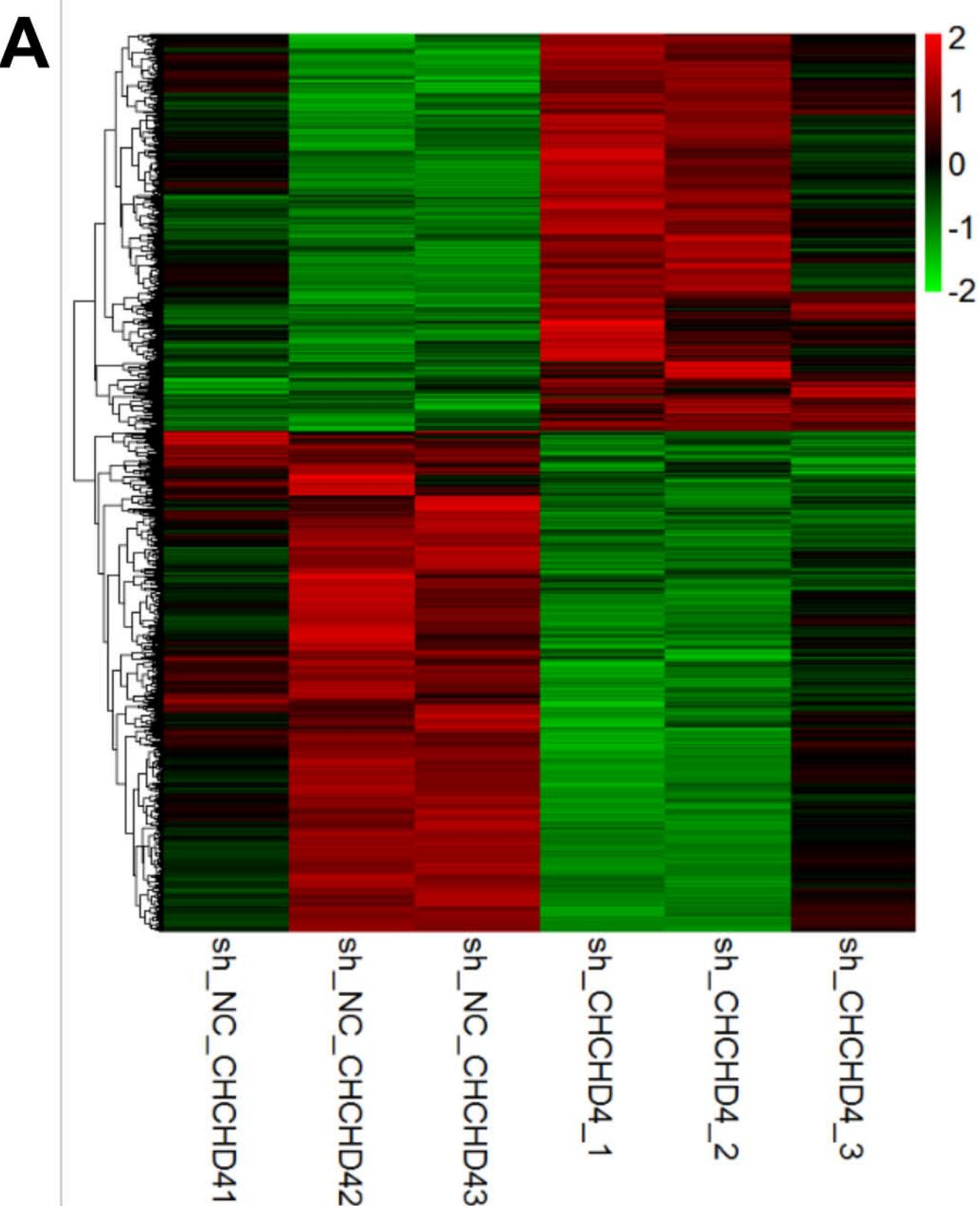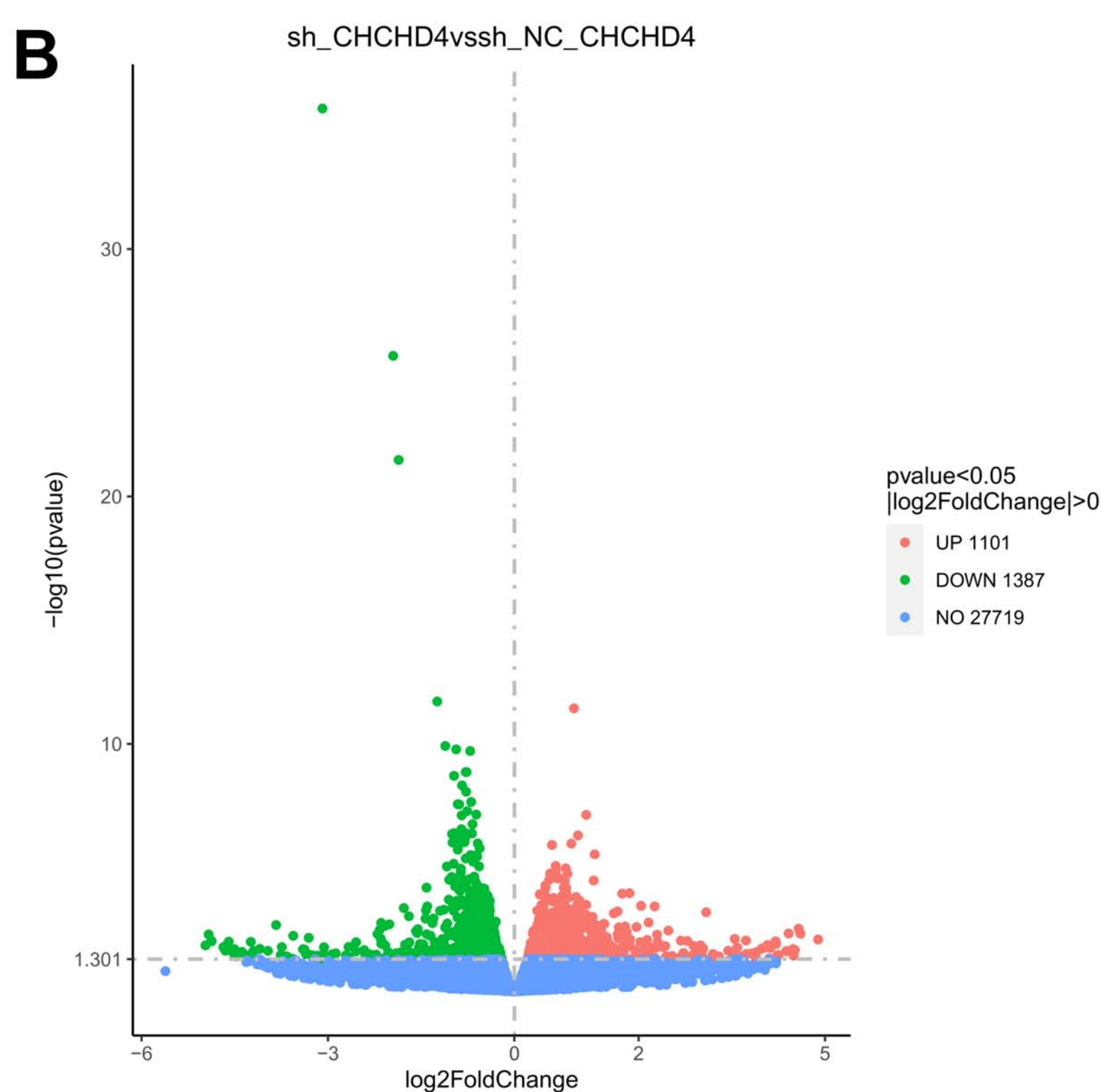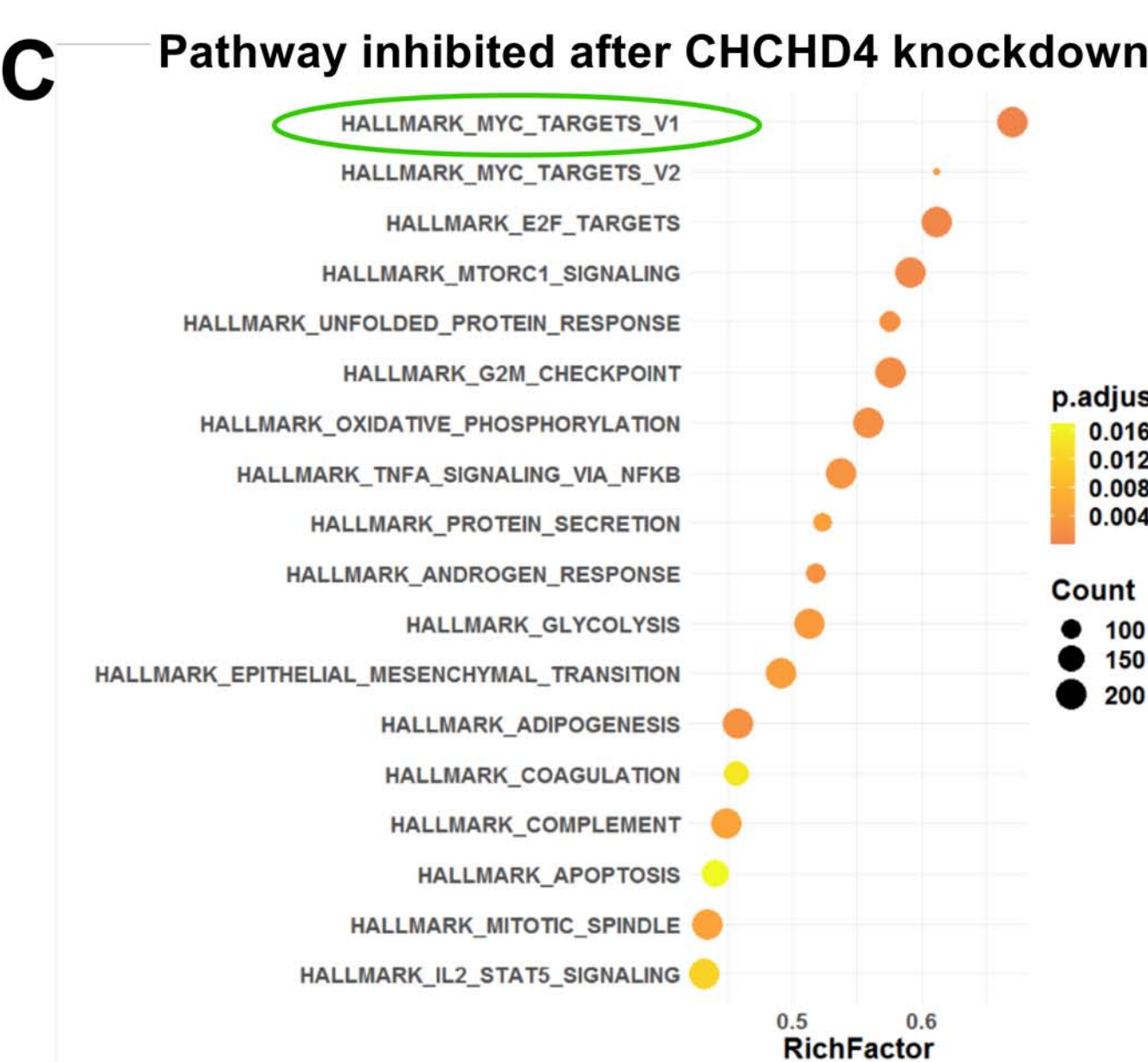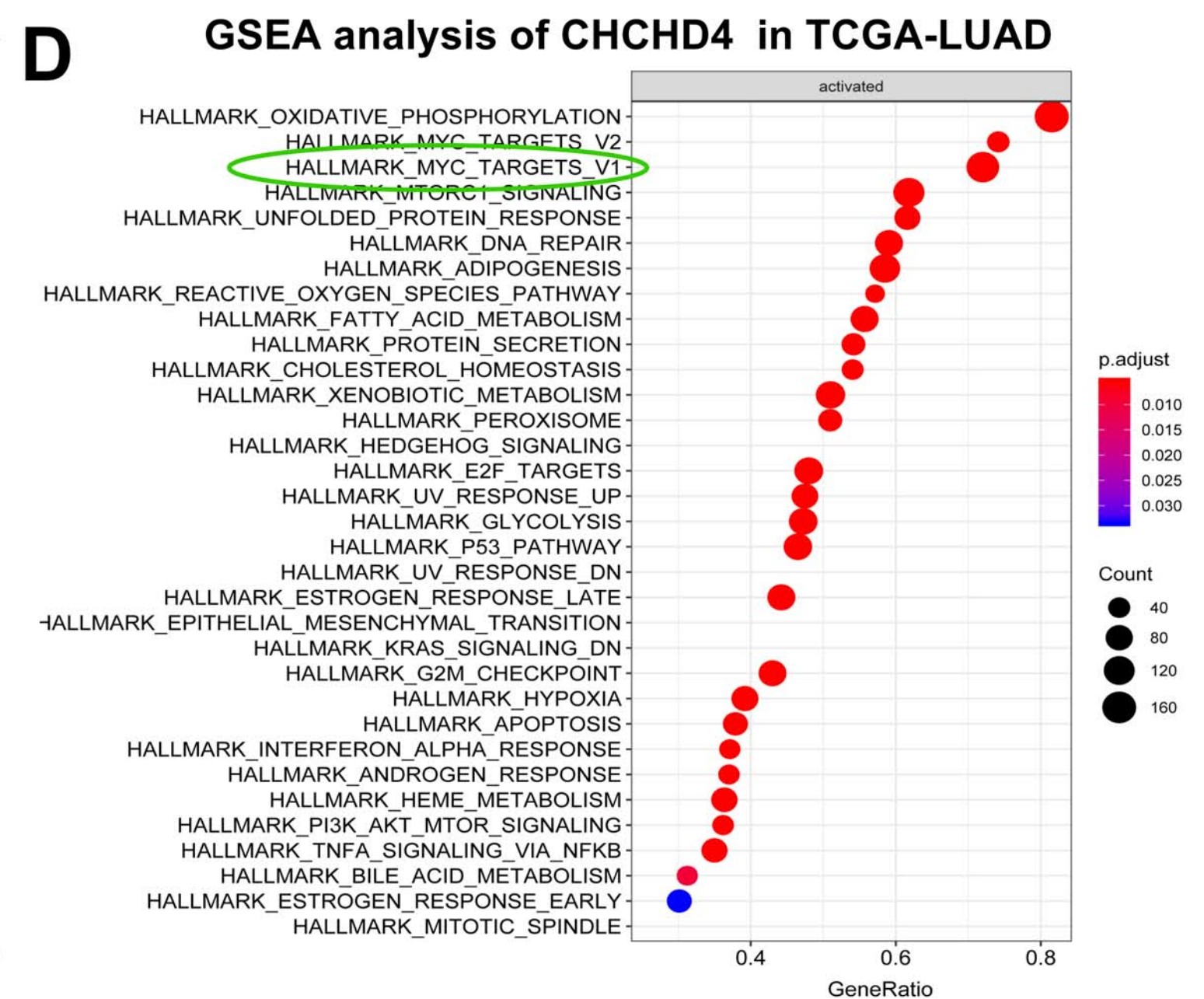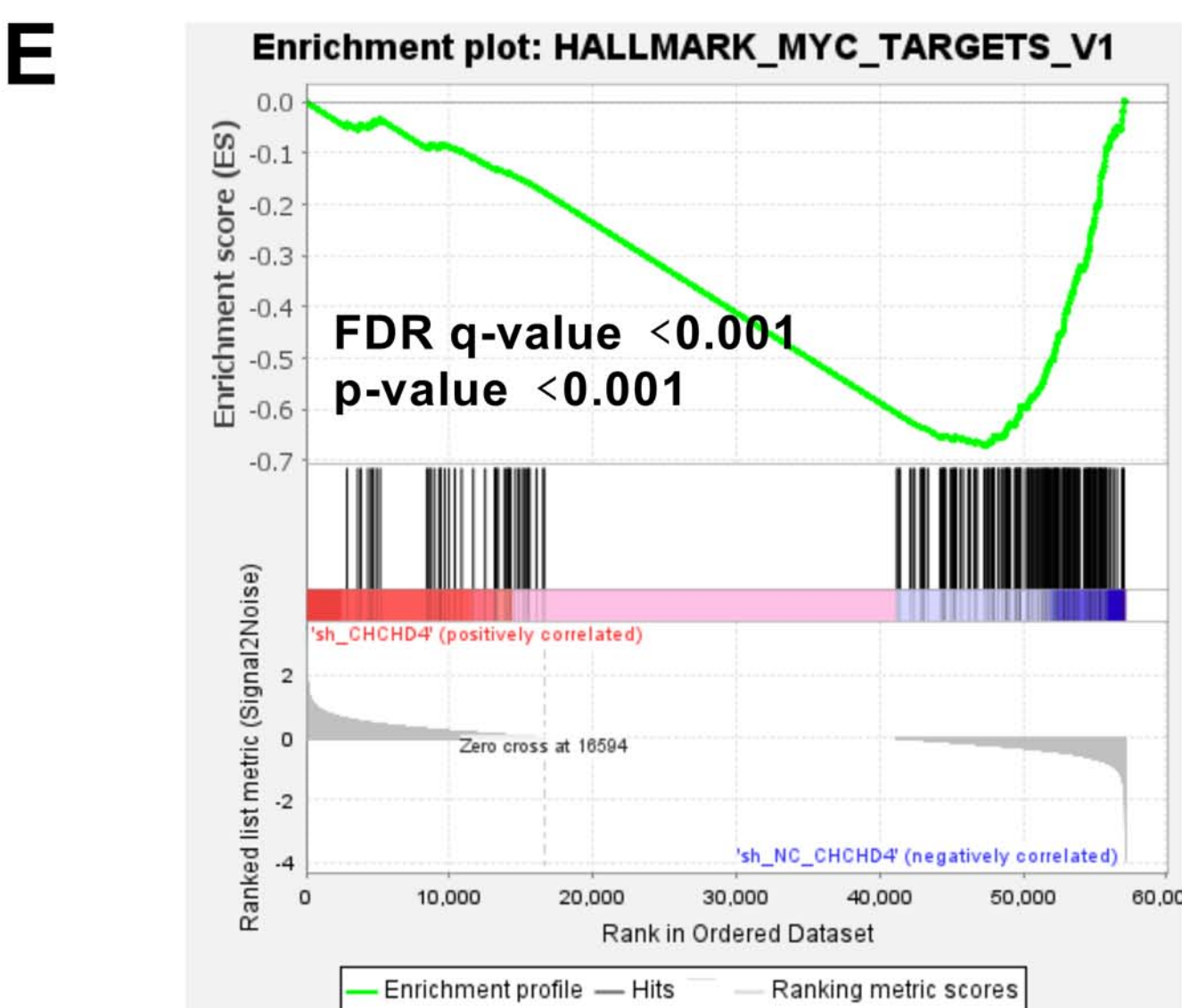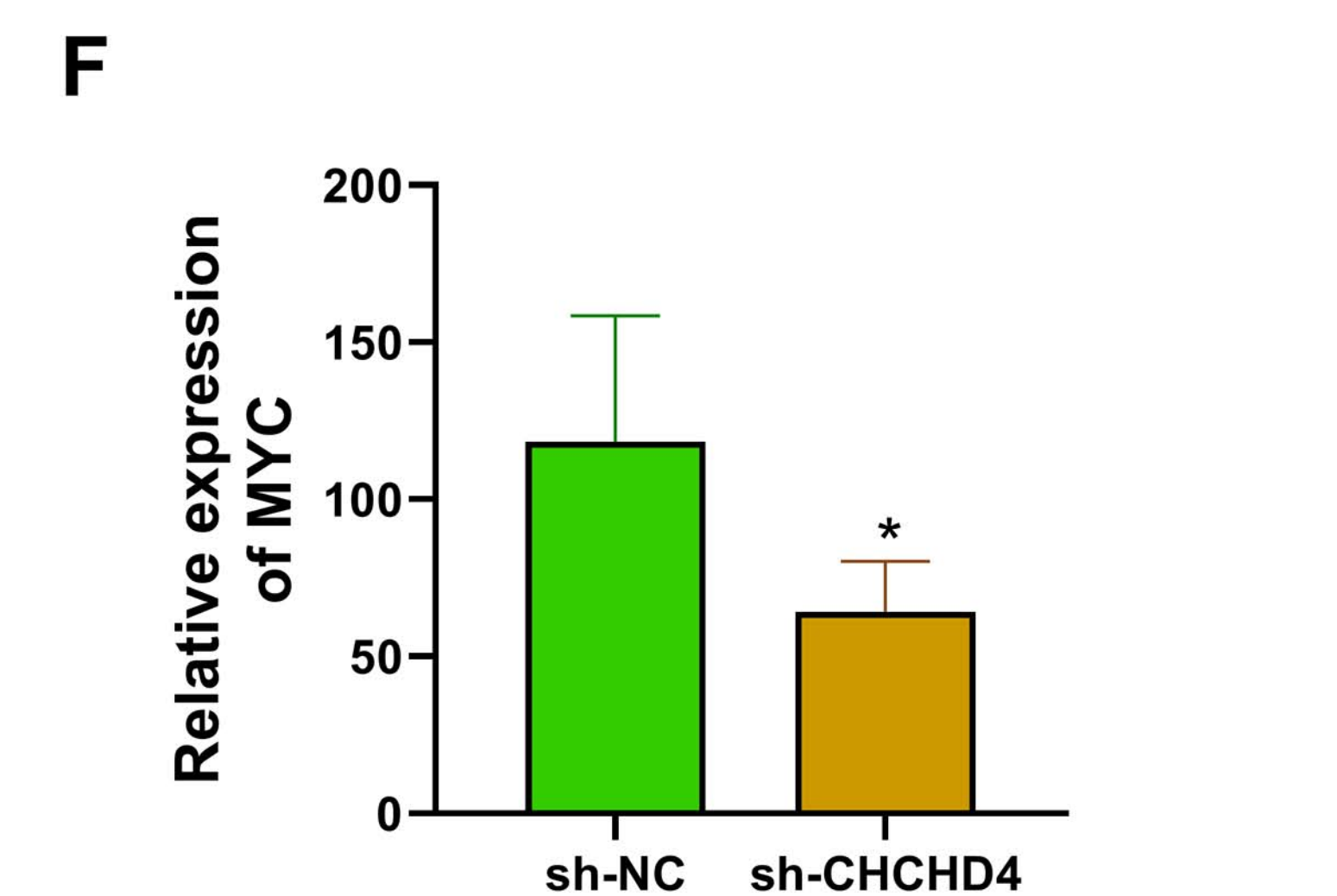

Supplement: Supplementary file 4 — Supplementary material 4: Figure S4. the transcriptome mRNA sequencing results of A549 cells transfected with sh-NC and sh-CHCHD4 respectively. (A and B) The transcriptome mRNA sequencing of A549 cells transfected with sh-CHCHD4 was sequenced by Novogene. (C) The data of GSEA analyses confirmed that CHCHD4 positively corelated with MYC signalling in A549 cells based on the results of transcriptome mRNA sequencing. (D) The data of GSEA confirmed that CHCHD4 positively corelated with MYC signalling in LUAD based on the TCGA-LUAD data. (E) MYC pathway was significantly inhibited after knockdown of CHCHD4 according to sequencing results. (F) The transcriptome mRNA sequencing results showed that MYC expression in sh-CHCHD4 group was significantly reduced. *P < 0.05 [file 12672_2022_600_MOESM4_ESM.pdf]
